# Hyponatremia after neuroendoscopic skull base tumor surgery: Clinical characteristics and nursing management

**DOI:** 10.3389/fsurg.2022.994102

**Published:** 2022-11-04

**Authors:** Yanjun Yang, Chunmei Lv, Jing Zhang, Yuanli Zhao

**Affiliations:** ^1^ Department of Neurosurgery, Peking University International Hospital, Beijing, China; ^2^ Department of Outpatient, Peking University International Hospital, Beijing, China

**Keywords:** hyponatremia, skull base tumor, neuroendoscopy, complication, nursing management

## Abstract

**Purpose:**

The current study was conducted to explore the clinical characteristics of hyponatremia after neuroendoscopic skull base tumor resection, and to summarize the nursing experience and provide insight for nursing management.

**Methods:**

In total, we enrolled 181 patients who underwent neuroendoscopic resection of skull base tumors in the Department of Neurosurgery of our hospital from 2016 to 2021. The patients' general data and parameters, including blood sodium level, polyuria, and other symptoms in different periods after surgery, were retrospectively reviewed.

**Results:**

Forty-four patients developed hyponatremia after Surgery. The total incidence of hyponatremia was 24.30%, including 38 cases of mild hyponatremia and 6 cases of moderate and severe hyponatremia. Most cases of moderate and severe hyponatremia occurred 6 days after surgery. The incidence of hyponatremia varied in different pathological types and periods in patients undergoing skull base tumors. After standardized sodium supplementation, water restriction, and urine volume control, hyponatremia was corrected in all patients, and no osmotic demyelination syndrome (ODS) and nursing-related events occurred.

**Conclusion:**

Secondary hyponatremia after neuroendoscopic resection of skull base tumors can occur in various time periods after surgery. Early monitoring of manifestations and standardized intervention are thus necessary for clinical nursing practice to timely correct hyponatremia and avoid demyelination.

## Introduction

Skull base tumor is a life-threatening condition that originates from the skull base and its adjacent tissue structures. The disease develops from the cranial to the extracranial or from the cranial to the intracranial compartments, passing through the skull base fissure, and growing after destroying the skull base bone (1). Currently, the most effective treatment for skull base tumors is surgical resection. However, traditional craniotomy is traumatic, and the pulling or touching of the surrounding brain tissue during the surgery may cause serious complications, such as hearing loss and facial paralysis. Neuroendoscopy can directly reach the skull base through the natural channel of the nasal cavity. Its application in skull base tumor resection has the advantages of small trauma, less bleeding, and rapid postoperative recovery. It has gradually been recognized by clinicians and patients, with wide applications in clinical practice (2, 3). Due to the complex tissue structure surrounding skull base tumors, it is still inevitable to cause damage to the pituitary gland, hypothalamus, and other critical neuroendocrine regulatory centers when the tumor is resected by neuroendoscopy. Based on previous studies, the incidence of delayed hyponatremia after the neuroendoscopic resection of skull base tumor was 15.34%–19.8% (4, 5), which is closely related to the abnormal secretion of antidiuretic hormone (6) and brain natriuretic peptide, and the sympathetic nerve function. It often causes brain edema, mental abnormalities, and even death of patients. During treatment, inappropriate correction of hyponatremia may result in complications such as water electrolyte disorder (7). Moreover, very fast correction of hyponatremia can cause osmotic demyelinating syndrome (ODS) (8). Standardized assessment of patients and detailed nursing management to control or reduce the occurrence of hyponatremia are challenging for doctors and nursing staff (7). The purpose of this study was to retrospectively analyze the characteristics of hyponatremia in patients with skull base tumors who underwent neuroendoscopic resection in our hospital and to summarize the nursing experience of hyponatremia, in an attempt to provide basic evidence and insight for the postoperative rehabilitation of patients and to improve the quality of clinical practice.

## Materials and methods

### Clinical background

We enrolled 181 patients who underwent neuroendoscopic resection of skull base tumors in the Department of Neurosurgery of our hospital from March 2016 to April 2021. The participants included 95 males and 86 females, with a median age of 46 years (46.8 ± 14.8). The inclusion criteria were as follows: (1) patients with skull base tumors confirmed by imaging and pathology; (2) neuroendoscopic tumor resection. The exclusion criteria were as follows: (1) patients diagnosed with co-existing tumors in other organs; (2) patients with hyponatremia before surgery. There was no statistical difference in baseline data between the two groups, as shown in Table 1.

**Table 1:** Clinical data of two groups.

Group	Case	Sex		Age (year)		Tumor type		Degree of tumor resection	
		Male	Female	≤46	>46	Pituitary adenoma	others	Total resection	Subtotal resection
Hyponatremia group	44	22	22	29	15	37	7	27	17
Normonatremia group	137	73	64	63	74	129	8	103	34
*X* ^2^		0.144		5.289		4.443		3.143	
*P*		0.704		0.021		0.035		0.076	

### Treatment method

#### Surgical intervention

All patients underwent neuroendoscopic skull base tumor resection after general anesthesia, and the specimens were timely collected for examination to determine the pathological classification. Skull base reconstruction was performed during the surgery.

#### Treatment of hyponatremia

##### Oral sodium supplementation

For sober patients, doctors guided them to eat more sodium-rich foods (such as pickled vegetables and pickled foods) or drink warm saline water (7). Oral salt has a poor taste and is irritating to the oral cavity and gastrointestinal tract, increasing the amount of drinking water, which results in poor compliance of patients. Therefore, we guided the patients to take oral salt capsules (salt into empty capsules) with meals, to reduce the stimulation of the patient's taste and sodium salt excretion caused by transient blood volume expansion following intravenous rehydration. The dosage of salt capsules was determined according to the blood sodium level of patients, generally 12 g/day, 3–4 times.

##### Intravenous sodium supplementation

Intravenous sodium supplementation was given according to the doctor's instructions, based on the urine osmotic pressure and actual condition of patients. The amount of sodium to be supplemented was calculated according to the formula: sodium to be supplemented (mmol) = (normal blood sodium (mmol/L)—measured blood sodium (mmol/L)) * body weight (kg) * 0.5 (female) or 0.6 (male) (9). An increase in blood sodium >10 mmol/L should be avoided in the first 24 h, and then <8 mmol/L every 24 h. The rate of sodium supplementation should not be very fast. The rising rate of blood sodium should be maintained below 0.7–1.0 mmol/(L·h), and not exceed 12 mmol/L in 24 h. According to the calculation that 17 mmol sodium is equal to 1 g sodium chloride, in the present study, 60–80 ml of 10% sodium chloride was generally added to 500 ml of 0.9% sodium chloride solution; the infusion pump was used to control the drip rate at 140–145 ml/h, and the infusion was completed within 4 h. After the target level of blood sodium was reached, the sodium supplementation was continued according to the urine volume and urine sodium level. The total amount of urine sodium excreted in the last 24 h plus the physiological requirement was supplemented daily until the blood sodium level became stable (10, 11).

##### Control of urine volume and restricted intake

After the surgery, doctors and nurses guided and assisted the patient to accurately measure and record their urine volume, and actively asked them whether there was any increase in urine volume, especially the urine volume per hour. If the urine volume per hour is ≥200 ml for 2 consecutive hours, it is necessary to inform the doctor in time (12). According to the doctor's instructions, 0.1 mg desmopressin acetate was given until the urine volume was controlled. If necessary, urinary sodium and 24-hour urine osmotic pressure was monitors, and water restriction measures were implemented according to the doctor's advice.

### Evaluation indicators and data collection

#### Diagnostic criteria of hyponatremia

Mild hyponatremia: 130–135 mmol/L; Moderate to severe hyponatremia: <130 mmol/L (13).

#### Nursing related events

Nursing related events were collected during the hospitalization, such as falling down and falling off the bed.

### Statistical analysis

Excel 2007 and SPSS 23.0 were utilized for data collection and statistical analysis. Quantitative data were expressed as means and standard deviation, and qualitative data were expressed as frequency and percentage (%) and compared using the chi square test. A *p* < 0.05 indicated statistical significance.

## Results

### Incidence of postoperative hyponatremia in patients with different types of skull base tumors

In this study, the incidence of hyponatremia after growth hormone type pituitary adenoma surgery was the lowest, with the earliest occurrence time and fastest correction time. The incidence, occurrence, and correction time of postoperative hyponatremia in patients with different types of skull base tumors are shown in Table 2

**Table 2:** Occurrence and correction of postoperative hyponatremia in patients with different types of skull base tumors.

Tumor classification	Gonadotropic pituitary adenoma	Adrenergic pituitary adenoma	Prolactin type pituitary adenoma	Growth hormone type pituitary adenoma	Multihormone cellular pituitary adenoma	Pituitary adenoma without endocrine function	Unclassified pituitary adenoma	Other types besides pituitary adenoma
Case	39	28	14	29	24	26	6	15
Cases of hyponatremia	8	5	6	2	9	4	1	9
Incidence of hyponatremia (%)	20.5	17.9	42.9	6.9	37.5	15.4	16.7	60
Time of hyponatremia (day)	5.75 ± 4.59	4.60 ± 3.65	6.00 ± 3.10	1.50 ± 0.71	8.25 ± 5.78	7.75 ± 3.30	0	4.25 ± 3.85
Correction time (day)	4.75 ± 3.37	8.80 ± 12.03	7.50 ± 4.09	3.50 ± 2.12	7.25 ± 6.63	4.50 ± 2.38	2.00	6.50 ± 4.34

### Incidence of postoperative hyponatremia in patients with skull base tumors at different stages

Some studies depicted that hyponatremia occurring 3 days after surgery is late-onset hyponatremia (5), and other studies suggested that hyponatremia occurring 5–7 days after surgery is late-onset hyponatremia (14). In our study, 33 patients (75%) developed hyponatremia 3 days after surgery, including all patients with moderate to severe hyponatremia. The incidence of postoperative hyponatremia in patients with skull base tumors at different stages is shown in Table 3.

**Table 3:** Incidence of hyponatremia among patients in different periods.

Time of postoperative hyponatremia (day)	Mild hyponatremia (case)	Moderate to severe hyponatremia (case)
<3	11	0
3–6	11	1
>6	16	5

### Urine volume control

In this study, there were 98 patients with polyuria after surgery, of which 96 were orally or intravenously treated with desmopressin acetate, and the effect of urine volume control was good.

### Nursing-related events

All patients had no nursing-related events such as scald, fall and bed fall during their stay in the hospital.

## Discussion

### The incidence of hyponatremia varied in patients with skull base tumors in different pathological types and different periods

According to some earlier studies, the incidence of delayed hyponatremia after surgery for non-secretory pituitary adenoma and adrenocorticotropic cell adenoma was higher than that of other groups. The clinical symptoms of patients with moderate to severe hyponatremia could be improved within approximately 2 days when treated with hypertonic saline and hydrocortisone (15). In this study, the incidence of hyponatremia after growth hormone type pituitary adenoma surgery was the lowest, with the earliest occurrence time and fastest correction time, which was thought to be associated with the routine hydrocortisone supplementation in the early postoperative period. Based on our study, hyponatremia occurred in all patients from 0 to 30 days after surgery, suggesting that the blood sodium level of patients should be closely monitored throughout the perioperative period and should be an important part of the follow-up after discharge.

### Pay close attention to the changes in blood sodium value and select the appropriate sodium supplementation approach to correct hyponatremia

Several studies (16, 17) have shown that even mild hyponatremia can have an impact on the prognosis of patients. In nursing management, the nursing of patients with mild hyponatremia should not be neglected, but close attention should be paid to their changes. Dynamic monitoring of 24 h urinary sodium and blood electrolyte levels is an important method for observing the effect and timely adjusting the strategy of sodium supplementation. Blood samples are collected 4 h after sodium supplementation (13), and the collection should be avoided when sodium-containing drugs are infused. If the patient is receiving liquid infusion into one limb, the other limb should be selected for collection. It is prohibited to collect blood samples from Peripherally Inserted Central Catheter(PICC) catheterization or other deep vein catheterization ends and through the peripheral indwelling needle to avoid the influence of residual sealing solution in the pipeline on the test results. If it is needed to puncture the indwelling needle and collect blood at the same time, avoid passing saline through the tube before puncture. When intravenous infusion of highly concentrated saline is used, an infusion pump is applied to control the speed of sodium supplementation. This method does not only achieve a constant infusion speed, but also prevents the patients or others from adjusting the drip speed by themselves or forgetting to adjust the drip speed when changing the liquid and consequently cause adverse consequences due to rapid infusion. Different brands of equipment indeed have differences in flow rate and accuracy (18). It is therefore recommended to select an infusion pump of the same brand and use the matching infusion device to calibrate the infusion pump on time to ensure reliable quality and accurate data of the infusion pump. In this study, the patients did not have severe complications such as osmotic demyelination syndrome (ODS) due to the rapid rise of blood sodium concentration.

### Control of urine volume and restriction of intake are significant steps to correct hyponatremia

Due to the special location of the skull base tumor, the pituitary stalk and hypothalamus may be interfered with during the surgery, and the secretion of antidiuretic hormone, polyuria, and disturbance of water and salt metabolism may occur, resulting in hyponatremia. Most of the symptoms are transient, which could be managed with antidiuretic analogue treatment (5). Timely supplementation of desmopressin acetate can reduce sodium loss. In this study, during the use of desmopressin acetate, the patients' urine color and urine volume were accurately assessed. Once the urine volume was controlled, the drug could be stopped. The family members were guided to use unified and standard measuring tools to measure and record the drinking water volume with a graduated measuring cup, and the water content of food was assessed and recorded by the nurses. The same graduated measuring cup was utilized to calculate the volume, especially the urine volume. On the other hand, the use of disposable drainage or urine bags as measuring tools should be avoided. Bedridden patients were assisted to discharge their urine to the urinal, and then use the measuring cup for measurement. The entry and exit record sheet was placed beside the bed for easy recording. The patients were reminded not to directly discharge their urine into the toilet for estimation. Burke et al. reported that the incidence of hyponatremia in patients with pituitary adenomas can be significantly reduced by strictly limiting the daily water intake to 1,000 ml one week after surgery (19). Therefore, the patients were advised to avoid drinking a lot of water and to control the drinking water volume within 1,000 ml/day after surgery.

### Pay attention to the bedside handover and the patient's complaints and performance, and jointly ensure the safety of patients

The clinical symptoms of hyponatremia vary according to its severity and the rate of blood sodium decline (7). The clinical manifestations of early hyponatremia lack specificity. During the critical process of shift handover, doctors and nurses should closely enquire and assess whether patients with hyponatremia have symptoms such as weakness of both lower limbs, poor appetite, dizziness, and inattention; hang high-risk tips for falls at the bedside; and instruct their families to pay attention to the patients' emotions. The patients should be accompanied by their families to timely inform abnormalities and improve safety management. Nursing-related events such as falling, scalding, and bed falling should be avoided. After sodium supplementation, blood samples should be taken on time for recheck. The results of blood sodium re-examination should be closely monitored to timely inform the doctor and adjust the sodium supplementation scheme according to the changes in blood sodium value. Oral sodium supplementation and dietary sodium supplementation are also considered important methods to supplement sodium. In our study, nurses chose the method of putting salt into capsules, which provides little stimulation on oral taste and accurate sodium supplementation. The acceptance of oral sodium supplementation was improved, reflecting the humanistic spirit of nursing.

## Conclusions

In summary, we showed that patients who underwent endoscopic resection of skull base tumors were prone to hyponatremia at different times after surgery. Based on the characteristics of its occurrence and development, nurses should focus on postoperative observation, accurate assessment, and medication administration to identify the causes of hyponatremia through observation. Moreover, the principles, methods, and approaches of sodium supplementation should be mastered to complete the sodium supplementation treatment in a standardized manner, so that hyponatremia can be corrected. In clinical nursing practice, efforts are made through propaganda and education to guide the process of sodium supplementation and to evaluate the effect. Nurses should be involved in the whole management process of patients with hyponatremia and truly implement the details of nursing measures, so that patients could avoid complications, ensure safety, and recover as soon as possible.

## Data Availability

The original contributions presented in the study are included in the article/Supplementary Materials, further inquiries can be directed to the corresponding author/s.
